# 70th International Congress of the European Society for Cardiovascular and Endovascular Surgery and 7th International Meeting on Aortic Diseases

**DOI:** 10.1055/s-0042-1750295

**Published:** 2022-06-10

**Authors:** John A. Elefteriades, Bulat A. Ziganshin

**Affiliations:** 1 AORTA Journal Editorial Office, Aortic Institute at Yale New Haven Hospital, Yale University School of Medicine, New Haven, Connecticut

AORTA is proud to publish the abstracts from the 2022 joint IMAD (International Meeting on Aortic Diseases) and ESCVS (European Society for Cardiovascular Surgery) meeting in Liege, Belgium.

To assure availability for the meeting, these abstracts are being published in original form, without proof reading or copy editing.

**IMPORTANT NOTE:** *AORTA is pleased to receive full manuscripts corresponding to these abstracts for expedited review and publication.*

